# Targeted Enrichment and Characterization of Diester Diterpenoid Alkaloids in *Aconitum* Herbs Using Gas–Liquid Microextraction Coupled with High-Resolution Mass Spectrometry

**DOI:** 10.3390/molecules30194029

**Published:** 2025-10-09

**Authors:** Yijun Wang, Ceyu Miao, Junxian Wu, Yutong Hua, Xiang Li, Liping Kang, Zidong Qiu

**Affiliations:** 1 State Key Laboratory for Quality Ensurance and Sustainable Use of Dao-di Herbs, National Resource Center for Chinese Materia Medica, China Academy of Chinese Medical Sciences, Beijing 100700, China; wangyijun2025@163.com (Y.W.); wjx_cdtcm@126.com (J.W.); honey1998@yeah.net (Y.H.); m18222616303@163.com (X.L.); 2 Graduate School, Tianjin University of Traditional Chinese Medicine, Tianjin 301617, China; 3 Shandong Center for Food and Drug Evaluation & Inspection, Jinan 250014, China; miaoceyu@163.com

**Keywords:** diester diterpenoid alkaloids, gas–liquid microextraction, direct mass spectrometry, *Aconitum*, trace analysis

## Abstract

Diterpenoid diester alkaloids (DDAs) are the primary toxic constituents in aconite herbs, while also being the key pharmacologically active components. Consequently, establishing rapid enrichment and highly sensitive analytical methods for DDAs is of critical importance. Herein, we developed and constructed a gas–liquid microextraction (GLME) device, which enables the rapid and selective enrichment of DDAs from complex matrices. The enriched extract can be directly analyzed by high-resolution Orbitrap mass spectrometry without requiring any further pretreatment. A comparative analysis of six commonly used *Aconitum* herbs medicines and their processed derivatives was conducted. Notably, GLME enhanced the mass spectrometric signals of DDAs by 3–4 orders of magnitude, facilitating the identification of 27 alkaloids, including 3 potential new compounds (15-Ethyl-13-deoxyanhydroaconitine, 13-Hydroxy-15-ethylanhydroaconitine and 8-eicosapentaenoic-benzoylmesaconine). It was found that among the tested samples, the DDAs response intensity of raw Caowu was the highest, and the DDA signals decreased significantly after processing. This result chemically validates the detoxification efficacy of traditional methods. The proposed GLME-MS strategy has the advantages of being green, economical, easy to operate, and highly selective (>1000-fold), which provides a technical reference for the rapid detection, safety assessment, and quality control of *Aconitum* herbs.

## 1. Introduction

Aconite-based traditional Chinese medicine (TCM) has a long history of application within China’s TCM practice [1,2,3]. Representative herbs such as Chuanwu (the mother root of *Aconitum carmichaelii*), Caowu (the root tuber of *Aconitum kusnezoffii*), and Fuzi (the processed daughter root of *Aconitum carmichaelii*) are widely used clinically [4]. Modern pharmacological studies have shown that these herbs possess significant pharmacological activities, including anti-inflammatory, analgesic, and cardiotonic effects, among others [5,6,7,8]. Their pharmacodynamic material basis is primarily attributed to various alkaloid components [9]. *Aconitum* alkaloids are primarily classified into diester, monoester, and non-ester aconitine types based on their structural characteristics. Among these, diester diterpenoid alkaloids (DDAs) can be further categorized into acetates and fatty acid esters. Acetates represent the most common and typical DDAs, whereas fatty acid esters are often termed lipo-alkaloids. Toxicity varies significantly among these classes, with DDAs demonstrating the most acute toxicity. Their toxicity is estimated to be 200–500 times greater than that of monoester diterpenoid alkaloids and 2000–4000 times greater than that of non-ester alkaloids [10]. This high toxicity is closely related to the ester bonds at the C8 and C14 positions of their molecular structures [11]. Although clinically processed *Aconitum* herbs are commonly used to reduce toxicity, existing processing techniques still struggle to completely eliminate DDA residues. Notably, even trace amounts of DDAs can cause severe toxic reactions that are potentially life-threatening [12]. However, current pharmacopeia standards only use the total content of aconitine, mesaconitine, and hypaconitine as quality control indicators, failing to cover other highly toxic DDAs like yunaconitine. In fact, the wide variety of DDAs with differing toxicities means that relying on only a few components for evaluation may lead to an underestimation of potential safety risks. Therefore, establishing a rapid and sensitive method for the targeted enrichment and detection analysis of DDAs in aconite-based TCM is crucial for improving clinical medication safety standards and enhancing the quality control level of these herbs.

The efficient separation, enrichment, and detection of DDAs in aconite drugs have consistently posed a key technical challenge in the field of traditional Chinese medicine quality control. Current enrichment and pretreatment methods for these toxic components primarily rely on techniques such as solid-phase extraction (SPE) [13] and liquid–liquid extraction (LLE) [14]. SPE operates on interactions between liquid and stationary phases, achieving selective separation through specific hydrophobic, polar, and/or ion-exchange properties between the target analytes and the sorbent [15]. However, it is limited by cumbersome operational steps, time consumption, and high consumable costs [16]. Conversely, LLE separates and enriches analytes based on their differential partition coefficients between two immiscible liquids, yet it suffers from high organic solvent consumption and poor selectivity [17]. Recently, GLME technology [18,19] has garnered significant interest owing to its exceptional selective enrichment capabilities and cost-effectiveness. This technique integrates sample extraction, purification, and enrichment into a single efficient pretreatment step, markedly enhancing detection sensitivity through the selective concentration of trace target substances [20]. Its distinctive advantages include superior impurity removal capacity, effectively eliminating interferents such as proteins, polysaccharides, and salts [21]; operational simplicity; and economic–environmental benefits, as the process requires only water and an auxiliary gas [22,23], thereby substantially reducing organic solvent consumption.

Preliminary studies by our group revealed that GLME demonstrates a specificity enrichment factor exceeding 1000 for toxic DDAs, with negligible enrichment observed for monoester diterpenoid and non-ester alkaloids [20,24]. Building on this foundation, the present study developed a novel analytical approach by coupling GLME with direct infusion electrospray ionization mass spectrometry (DI-ESI-MS). Six representative *Aconitum* herbs, encompassing both raw and processed forms of Fuzi, Chuanwu, and Caowu, were selected for investigation. This established an efficient and targeted analytical strategy for the profiling of toxic DDAs in *Aconitum* herbs. This method successfully achieved the targeted enrichment and highly sensitive detection of numerous trace DDAs. A total of 27 alkaloids were identified, comprising 2 potentially novel traditional DDAs and 1 lipo-alkaloid. The remaining compounds included twelve known DDAs, ten lipo-alkaloids, and two other alkaloids. A systematic analysis was conducted to elucidate the distribution patterns and potential risks associated with these toxic components across the different herbs. This work provides a reliable technical framework that supports the development, utilization, and enhanced quality control of *Aconitum*-based TCMs and their toxic DDAs.

## 2. Results and Discussion

### 2.1. Construction and Optimization Conditions of GLME–Mass Spectrometry Device

**Figure 1:**
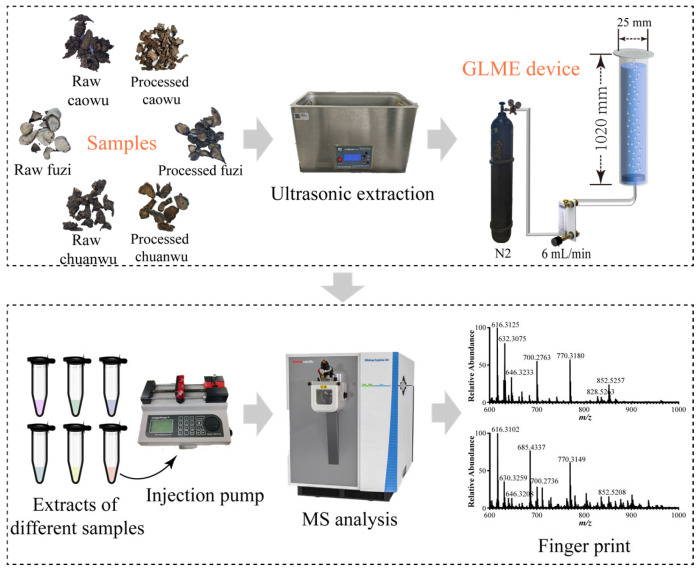
Gas–liquid microextraction–direct perfusion electrospray ionization mass spectrometry analysis process diagram (the pictures of medicinal materials and instruments were all taken by ourselves, and the final pictures were generated by WPS Office 12.1.0).

The main structure of the GLME device comprised a quartz glass tube (i.d. 25 mm; length 1020 mm) filled with the sample solution for analysis. A constant-temperature heating tape was wrapped around the external wall of the tube to maintain a stable temperature. An aperture (i.d. 7 mm) was fabricated at the bottom of the tube and fitted with a porous membrane (i.d. 100 μm). This membrane was connected to a nitrogen gas cylinder to generate micro-bubbles. The nitrogen flow rate was precisely controlled using a gas flow meter. A glass coverslip (24 mm × 32 mm) was positioned at an angle at the top of the quartz tube to collect micro-droplets generated by bursting bubbles. The device has the advantages of being highly efficient, economical, simple, and easy to operation. The use of green, cheap nitrogen and ultrapure water as the extraction carrier gas and solvent minimizes the chemical pollution of the environment or samples by traditional pretreatment methods. In addition, complex matrix solutions such as herbal extracts can be directly analyzed by DI-ESI-MS without pretreatment such as filtration and centrifugation after GLME (see the analytical workflow in Figure 1).

**Figure 2:**
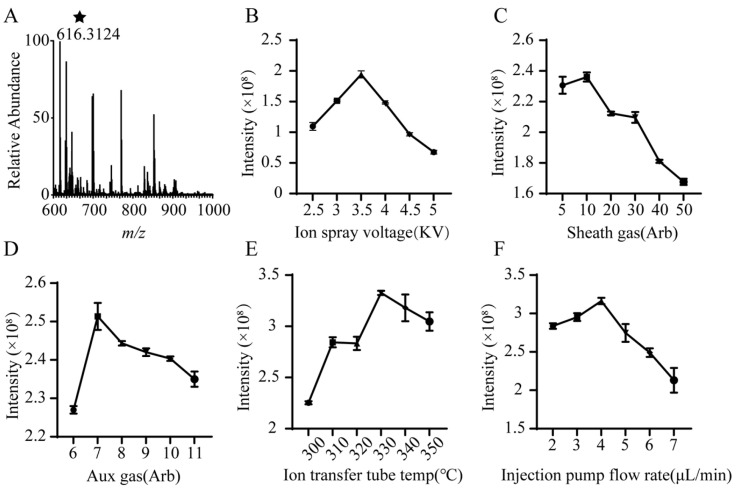
Optimization results of key mass spectrometry parameters (*n* = 3). (**A**) Finger print, (**B**) ion spray voltage, (**C**) sheath gas, (**D**) aux gas, (**E**) ion transfer tube temperature, (**F**) injection pump flow rate. (The key target analytes are marked with ★).

Subsequently, the developed GLME technique was applied to enrich trace DDAs in six distinct *Aconitum* materials. Prior to direct DI-ESI-MS analysis, key mass spectrometric parameters were systematically optimized to achieve optimal detection sensitivity, using the signal intensity of the target analytes as the indicator (Figure 2A). The results demonstrated that the ion spray voltage considerably influenced the signal intensity. As shown in Figure 2B, the optimal peak response was observed at 3.5 kV (NL = 1.94 × 10^8^), with deviations from this value resulting in signal attenuation. For the sheath gas, the optimal flow rate was 10 arbitrary units (arb) (NL = 2.36 × 10^8^), with further increases leading to a decline in signal intensity (Figure 2C). Optimization of the auxiliary gas flow rate yielded the highest signal intensity at 7 arb (NL = 2.51 × 10^8^), representing a 10.6% enhancement over the intensity at 6 arb (NL = 2.27 × 10^8^). However, exceeding this optimal flow rate resulted in signal reduction (Figure 2D). The ion transfer tube temperature also exerted a notable effect. The signal intensity increased with temperature from 300 °C to 330 °C, reaching a maximum (NL = 3.32 × 10^8^) at the latter temperature (Figure 2E). Finally, optimization of the sample flow rate revealed a relatively stable signal within the 2–4 μL/min range (NL = 2.83 × 10^8^–3.16 × 10^8^), peaking at 4 μL/min before undergoing a sharp decline at higher flow rates (Figure 2F).

### 2.2. Difference Analysis of Mass Spectrometry Before/After GLME

**Figure 3:**
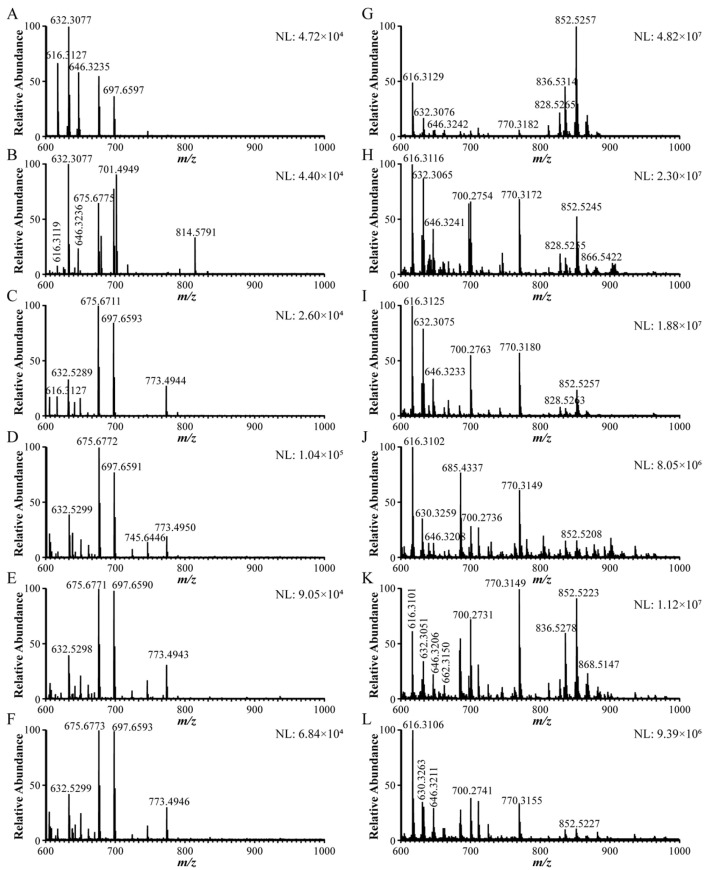
Comparison of mass spectra of 6 kinds of *Aconitum* herbs before and after gas–liquid microextraction. (**A**–**F**) Mass spectrometry results before enrichment of GLME, (**G**–**L**) mass spectrometry results after enrichment by GLME ((**A**–**L**) are raw Fuzi, raw Caowu, raw Chuanwu, processed Fuzi, processed Caowu, and processed Chuanwu).

Under the optimized MS conditions, the mass spectrometric fingerprints of Fuzi, Chuanwu, Caowu, and their processed products were compared before and after GLME treatment (Figure 3). The results demonstrated that GLME exhibited remarkable selectivity and enrichment capability for toxic DDAs within complex sample matrices. As shown in Figure 3A–F, the unenriched raw samples displayed complex background interference within the *m*/*z* 600–700 range (all signal intensities <1.04 × 10^5^) after blank subtraction, which completely obscured the characteristic peaks of the target alkaloids. In contrast, the GLME-treated samples (Figure 3G–L) showed a dramatic simplification of the spectra. Non-specific signals (e.g., at *m*/*z* 675, 697, 745, and 773) were effectively eliminated, whereas the [M+H]+ ions of target compounds, such as aconitine, became distinctly visible with significantly enhanced intensities (>10^6^ to 10^7^). This stark contrast conclusively demonstrates the high selectivity and powerful enrichment capacity of GLME for trace DDAs in complex matrices. Table S1 provides a detailed comparison of the MS response intensities for three highly toxic DDAs (hypaconitine, mesaconitine, and aconitine) across the six *Aconitum* herbs before and after GLME. The signals for the target compounds were enhanced by 2 to 4 orders of magnitude following GLME; for instance, the signal for hypaconitine in Caowu increased by approximately 7800-fold. Notably, the signal for aconitine, which was very weak or undetectable prior to enrichment, was dramatically enhanced after enrichment, elevating its concentration from undetectable levels to, for example, 2.61 × 10^6^ in processed Chuanwu. Collectively, these findings robustly validate the highly selective enrichment capability of GLME for trace-level, highly toxic DDAs in *Aconitum* herbal medicines.

Currently, the mechanism of selective enrichment and background interference removal of GLME can be mainly attributed to the surface activity-dominated molecular competition mechanism [25,26]. Specifically, during the bubble rising phase, the enriched components undergo dynamic distribution at the gas–liquid interface. In this process, organic molecules with high surface activity, due to their strong interfacial affinity, preferentially adsorb onto the bubble surface, thereby significantly enriching the interfacial phase; meanwhile, substances with low surface activity (such as most inorganic salt ions) are excluded in the interfacial competition, gradually moving away from the interfacial area and re-entering the solution, ultimately achieving efficient exclusion of background interferences [27]. In addition, based on the previous work of our research group [20], it was found that bubbles had directional and efficient enrichment of DDAs, but had almost no enrichment effect on monoester and non-ester aconitines, indicating that their enrichment tendency may also be related to the polarity of the compounds.

### 2.3. Identification of Chemical Constituents

**Figure 4:**
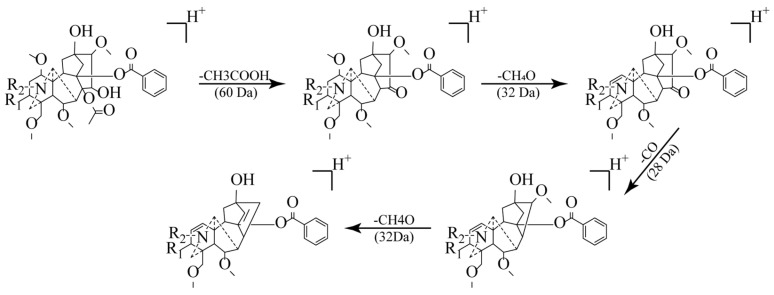
Elucidation of characteristic fragmentation pathways for DDAs.

Utilizing the GLME-enriched samples, characteristic ion peaks within the *m*/*z* 600–1000 range were systematically identified via an MS^2^ scan mode using a high-resolution mass spectrometer (Table 1). A total of 27 compounds were successfully identified based on accurate mass measurements (mass error < 5 ppm) and the interpretation of characteristic MS/MS fragmentation patterns. The identified compounds comprised twelve known DDAs (including key toxic constituents, e.g., hypaconitine, mesaconitine, and aconitine), two potentially novel DDAs, eleven lipo-alkaloids, and two other alkaloids. During structural elucidation, the DDAs exhibited characteristic fragmentation pathways (Figure 4): initially, the loss of acetic acid (CH_3_COOH, 60 Da) to yield the distinctive [M+H-60]^+^ ion, followed by subsequent losses of methanol (CH_3_OH, 32 Da) and carbon monoxide (CO, 28 Da), generating fragment ions such as [M+H-60-32]^+^ and [M+H-60-32-28]^+^ [22,23]. The structures of the 27 compounds are shown in Figure S1.

**Figure 5:**
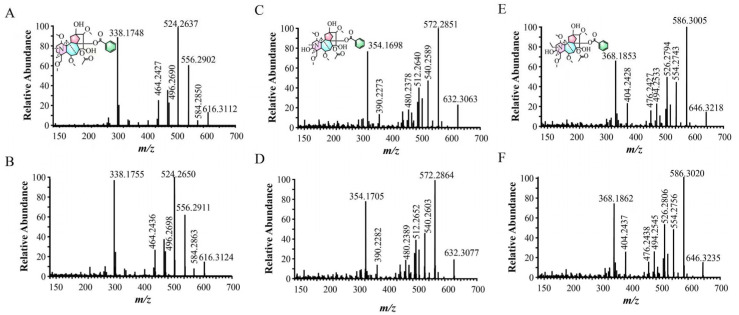
MS/MS comparison results of hypaconitine, mesaconitine, and aconitine in samples and reference standard. (**A**,**B**) Hypaconitine, (**C**,**D**) mesaconitine, (**E**,**F**) aconitine ((**A**,**C**,**E**) are the secondary mass spectra of the standard compound; (**B**,**D**,**F**) are the secondary mass spectra of the samples).

High-resolution mass spectrometry (HRMS) analysis of compound **1** revealed a quasi-molecular ion peak at *m*/*z* 600.3181 [M+H]^+^. The MS/MS spectrum exhibited characteristic fragment ions at *m*/*z* 540.2971 (corresponding to [M+H-C_2_H_4_O_2_]^+^, loss of acetic acid), 522.2844 ([M+H-C_2_H_4_O_2_-H_2_O]^+^), and 490.2584 ([M+H-C_2_H_4_O_2_-CH_4_O-H_2_O]^+^). Based on these fragmentation patterns and comparison with literature data [28,29], compound **1** was identified as delphinine. The [M+H]^+^ ions for compounds **4**, **6**, and **8** were observed at *m*/*z* 616.3132, 632.3082, and 646.3242, respectively. The MS/MS spectra of all three compounds displayed analogous fragmentation pathways: initial loss of acetic acid (C_2_H_4_O_2_, 60 Da) to form the [M+H-60]^+^ ion, followed by successive losses of 32 Da (CH_3_OH) and 28 Da (CO), yielding the [M+H-60-32]^+^ and [M+H-60-32-28]^+^ fragments, respectively. Subsequent direct comparison of the sample MS/MS spectra with those of authentic standards (Figure 5) confirmed identical fragmentation patterns and excellent agreement in fragment ion masses. Consequently, compounds **4**, **6**, and **8** were unambiguously identified as hypaconitine [30,31], mesaconitine [32], and aconitine [33], respectively. Compound **5** exhibited a quasi-molecular ion peak at *m*/*z* 630.3287 [M+H]^+^. Its MS/MS spectrum showed a fragmentation pattern analogous to the DDAs described above, generating characteristic ions at *m*/*z* 570.3072 ([M+H-60]^+^), 538.2811 ([M+H-60-32]^+^), and 510.2863 ([M+H-60-32-28]^+^). Based on comparison with literature data, compound **5** was characterized as 3-deoxyaconitine [34,35]. The MS/MS spectra of 11 known alkaloids identified by reference and online databases can be seen in Figure S2.

**Figure 6:**
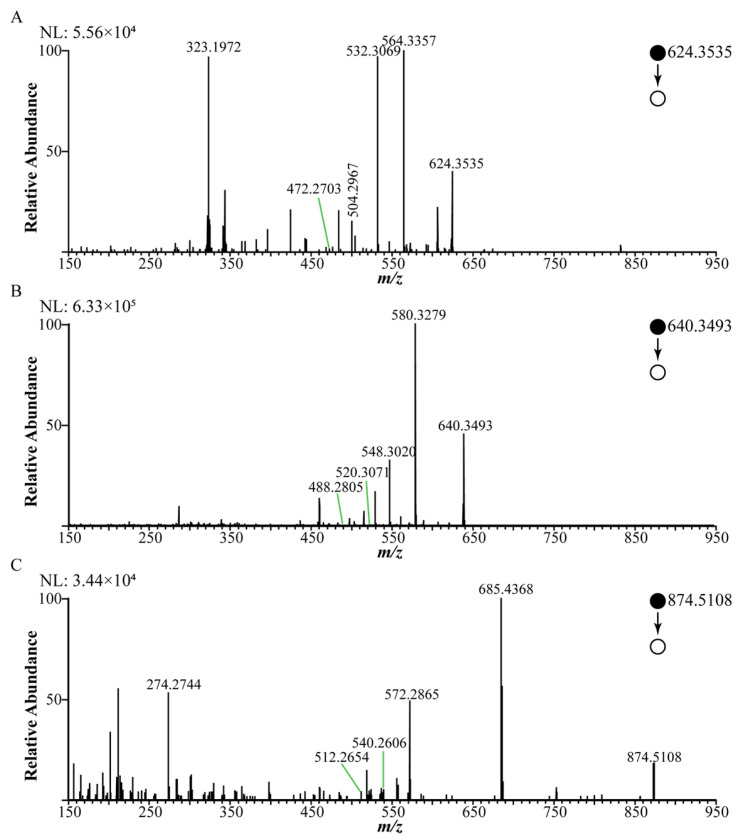
MS/MS spectra of three potential new compounds: (**A**) *m*/*z* 624.3535, (**B**) *m*/*z* 640.3493, (**C**) *m*/*z* 874.5108.

Compounds **13** and **14** exhibited a quasi-molecular ion peak at *m*/*z* 624.3541 and 640.3494, respectively (Figure 6A,B). Their MS/MS spectra displayed characteristic fragment ions corresponding to the loss of acetic acid at *m*/*z* 564.3335 and 580.3279 ([M+H-60]^+^). This observed fragmentation behavior is highly characteristic of DDAs and aligns with patterns previously reported [36]. Based on the established fragmentation mechanisms of DDAs [37], this neutral loss of 60 Da is rationalized by the elimination of acetic acid, likely involving the C8 acetoxyl group and a proton from the C15 position. Further fragmentation yielded successive losses consistent with methanol (32 Da) and carbon monoxide (28 Da), generating ions at [M+H-60-32]^+^ and [M+H-60-32-28]^+^, followed by an additional loss suggestive of a second methanol molecule ([M+H-60-32-28-32]^+^). These collective fragmentation patterns strongly support the classification of compounds **13** and **14** as diester diterpenoid alkaloids. A notable observation was the exact mass difference of 12.03 Da between compound **14** and compound **12**, which corresponds precisely to the mass difference expected for the replacement of a hydroxyl group by an ethyl group (12.03 Da). This suggests that compound **14** could be an ethyl-substituted analogue of compound **12**. Extensive searching of the literature and mass spectral databases (e.g., PubChem, HMDB) yielded no matches, preliminarily indicating that compounds **13** and **14** may be previously unreported novel DDAs. However, MS analysis provides only preliminary identification. Subsequent work will therefore focus on the targeted isolation of compounds **13** and **14**, followed by structural elucidation via techniques such as NMR spectroscopy and toxicity evaluation, to confirm their chemical structures and biological activities.

Compounds **17**–**27** were tentatively characterized as lipo-alkaloids. Their MS/MS spectra were dominated by neutral losses characteristic of fatty acid moieties. As summarized in Table 1, the predominant neutral losses observed corresponded to palmitic acid, linolenic acid, linoleic acid, and eicosapentaenoic acid. Notably, based on the literature and database comparisons, compound **27** is proposed to be a potentially novel lipo-alkaloid. It exhibited a protonated molecule [M+H]^+^ at *m*/*z* 874.5108 (Figure 6C). The MS/MS spectrum acquired in scan mode revealed a base peak at *m*/*z* 572.2865, resulting from a neutral loss of 302.2243 Da. This mass loss corresponds to the exact mass of eicosapentaenoic acid. Although lipo-alkaloids are typically present at low abundance [33], GLME enrichment dramatically enhanced their MS response, thereby providing a robust foundation for the detection and structural characterization of these trace constituents. The MS/MS spectra of 11 known lipid alkaloids identified by references and online databases can be seen in Figure S3. The follow-up study will carry out the targeted separation of compound **27** and investigate the impact of its unique fatty acid chain on the toxicity and activity of the compound.

**Table 1:** The mass spectrometry data of the 27 main compounds [M+H]^+^ identified in 6 types of *Aconitum* traditional Chinese medicines.

Type	No.	Formula	*m*/*z*	Error (ppm)	MS/MS	Compound Name	Reference
DDAs	**1**	C_33_H_45_NO_9_	600.3181	−2.3	540.2971, 522.2844, 490.2584, 480.2760, 458.2321, 448.2493	Delphinine	[28]
	**2**	C_32_H_43_NO_10_	602.2963	−0.7	542.2750, 510.2487, 482.2559, 450.2281	N-demethylhypaconitine	[38]
	**3**	C_34_H_47_NO_9_	614.3335	−1.8	554.3127, 522.2862, 494.2910, 462.2650	Chasmaconitine	[38]
	**4**	C_33_H_45_NO_10_	616.3132	−2.6	556.2919, 524.2656, 496.2707, 464.2443	Hypaconitine *	[30]
	**5**	C_34_H_47_NO_10_	630.3287	−2.2	570.3072, 538.2811, 510.2863, 478.2596	3-Deoxyaconitine	[35]
	**6**	C_33_H_45_NO_11_	632.3082	−2.7	572.2867, 540.2607, 512.2655, 480.2393	Mesaconitine *	[30,32]
	**7**	C_35_H_49_NO_10_	644.3447	−2.8	584.2872, 552.2608, 524.2659, 492.2394	Crassicauline A	[32]
	**8**	C_34_H_47_NO_11_	646.3242	−3.1	586.3019, 554.2763, 526.2813, 494.2551	Aconitine *	[33,39]
	**9**	C_33_H_45_NO_12_	648.3026	−1.7	588.2816, 556.2555, 528.2607, 496.2358	Beiwutine	[35]
	**10**	C_35_H_49_NO_11_	660.3391	−1.9	600.3167, 568.2913, 540.2607, 508.2346	Yunaconitine	[40]
	**11**	C_34_H_47_NO_12_	662.3187	−2.4	602.2976, 570.2712, 542.2758, 510.2499	10-Hydroxy aconitine	[39]
	**12**	C_34_H_45_NO_10_	628.3104	1.9	568.2889, 536.2631, 508.2680, 476.2421	Anhydroaconitine	[41]
	**13**	C_36_H_49_NO_8_	624.3541	−1.6	564.3349, 532.3078, 504.2972, 472.2705	15-Ethyl-13-deoxyanhydroaconitine	-
	**14**	C_36_H_49_NO_9_	640.3494	−2.2	580.3279, 548.3018, 520.3072, 488.2805	13-Hydroxy-15-ethylanhydroaconitine	-
Other	**15**	C_39_H_41_NO_11_	700.2765	−1.9	640.2556, 578.2397	Trifoliolasine E	[39,42]
	**16**	C_43_H_47_NO_12_	770.3180	−1.4	648.2811, 500.2074, 378.1707, 318.1491	(-)-1β,11a-diacetoxy-2α,13α-dibenzoyloxy-7β-hydroxy-15α-isobutanoyloxy-N-methyl-N,19-secohetisan-19-al	[39]
lipo-alkaloids	**17**	C_48_H_75_NO_10_	826.5501	−4.6	570.3065, 538,2805, 510.2856	8-palmitic-benzoyldeoxyaconitine	[43]
	**18**	C_48_H_76_NO_10_	828.5258	−0.2	572.2861, 540.2601, 512.2648	8-palmitic-benzoylmesaconine	[43]
	**19**	C_47_H_73_NO_11_	834.5154	−0.4	556.2901, 524.2646, 496.2703	8-linolenic-benzoylhypaconine	[43]
	**20**	C_49_H_71_NO_10_	836.5308	−0.1	556.2911, 524.2647, 496.2700	8-linoleic-benzoylhypaconine	[44]
	**21**	C_49_H_73_NO_10_	842.5416	−0.4	586.3015, 554.2753, 526.2807	8-palmitic-benzoylaconine	[43]
	**22**	C_48_H_75_NO_11_	848.5314	−0.8	570.3069, 538.2806, 510.2857	8-linolenic-benzoyldeoxyaconitine	[43]
	**23**	C_50_H_73_NO_10_	850.5478	−1.8	570.3065, 538.2806, 510.2857	8-linoleic-benzoyldeoxyaconitine	[43]
	**24**	C_50_H_75_NO_10_	852.5250	0.7	572.2859, 540.2600, 512.2650	8-palmitic-benzoylmesaconine	[44]
	**25**	C_49_H_73_NO_11_	866.5428	−1.8	586.3016, 554.2753, 526.2805	8-linoleic-benzoylaconine	[44]
	**26**	C_47_H_73_NO_10_	812.5318	−1.4	556.2913, 524.2653, 496.2701	8-palmitic-benzoylaconine	[44]
	**27**	C_51_H_72_NO_11_	874.5108	−1.0	572.2865, 540.2606, 512.2654	8-eicosapentaenoic-benzoylmesaconine	-

* Compared with reference standards.

### 2.4. Difference Analysis of Six Kinds of Aconitum Herbs

#### 2.4.1. DDAs

**Figure 7:**
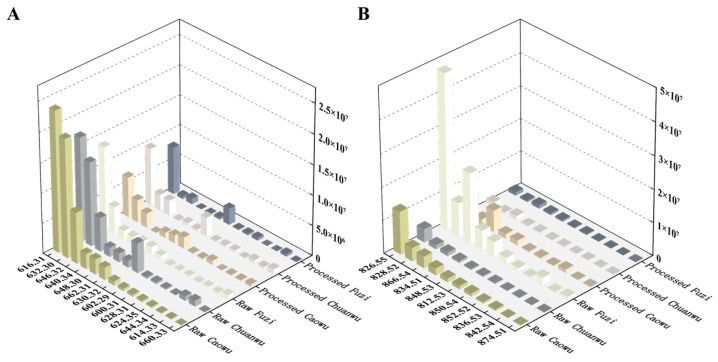
Mass spectrometry response difference analysis of characteristic components in raw/processed products of Fuzi, Caowu, and Chuanwu. (**A**) DDAs, (**B**) lipo-alkaloids.

DDAs constitute the primary toxic and pharmacologically active components in *Aconitum* herbs [45]. A comparison of MS response intensities (Figure 7) revealed substantial variations in DDA levels across the different herbs, with raw Caowu exhibiting a significantly higher response than the other samples. This distribution pattern aligns with the reported order of toxicity for these herbs [46]. As shown in Figure 6A, the processed Chuanwu, processed Caowu, and processed Fuzi exhibited a substantial decrease in DDA response intensity compared to their raw counterparts, with an average reduction of approximately 65%. This confirms the definitive detoxification effect of the traditional processing protocol. Furthermore, the DDA composition was notably altered by processing. While all three raw herbs contained 14 detectable DDAs, the versions showed a reduction: processed Caowu and processed Chuanwu contained 13 and processed Fuzi contained only 11. Despite this drastic decline in abundance, residual DDAs remained detectable at significant levels in the processed products. This finding carries significant implications for clinical practice: the use of processed products still mandates strict adherence to the traditional “decocting first and for a prolonged duration” (>1 h) protocol [47,48], complemented by necessary soaking procedures, to mitigate the potential risk posed by residual toxicity.

#### 2.4.2. Lipo-Alkaloids

Lipo-alkaloids represent a distinct class of diester diterpenoid alkaloid derivatives, characterized by the substitution of the C8 acetyl group in typical DDAs with various long-chain fatty acids (e.g., palmitic acid, linoleic acid). This structural modification is postulated to attenuate toxicity while preserving or potentially enhancing pharmacological activity [49,50]. As shown in Figure 6B, the MS response intensities for lipo-alkaloids were markedly higher in the raw herbs (Fuzi, Caowu, Chuanwu) than in their processed counterparts. This discrepancy is likely attributable to the hydrolysis of the C8 ester bond during processing, resulting in the degradation of lipo-alkaloids and their subsequent conversion to the corresponding monoester diterpenoid or non-ester alkaloids [44], thereby accounting for their reduced abundance. Based on these findings, future studies should prioritize systematically elucidating the contribution of lipo-alkaloids to the overall pharmacodynamic effects and their interactions with DDAs; establishing comprehensive quality control standards that include specific monitoring of lipo-alkaloids; and rigorously evaluating the potential toxicity risks of these compounds.

## 3. Materials and Methods

### 3.1. Chemicals and Materials

Reference standards for aconitine, mesaconitine, and hypaconitine (Table S2) were obtained from Beijing Beiterenkang Biotechnology Co., Ltd. (Beijing, China). The ultrapure water was sourced from Guangzhou Watsons Food and Beverage Co., Ltd., in Guangzhou, China. The raw and processed products of Aconiti Lateralis Radix Praeparata (Fuzi), Aconiti Kusnezoffii Radix (Caowu), and Aconiti Radix (Chuanwu) were sourced from Jiangyou Shenqi Chinese Herbal Medicine Planting Co., Ltd. (Sichuan, China). Among them, Fuzi, Chuanwu, and Caowu were the lateral roots of *Aconitum carmichaelii* Debx., the mother roots of *Aconitum carmichaelii* Debx., and the tuberous roots of *Aconitum kusnezoffii* Reichb., respectively, identified by Prof. Zhilai Zhan, and all the samples were stored in the National Resource Center for Chinese Materia Medica, China Academy of Chinese Medical Sciences, with the code WT2025001–WT2025018.

### 3.2. Parameters of GLME Device

Following a previously optimized protocol from our group [18], 5.0 mg of each sample powder was accurately weighed and transferred into 660 mL of distilled water. Subsequently, the mixtures underwent ultrasonic extraction for 20 min to yield extracts for each of the different samples. These extracts were then processed using a self-built gas–liquid microextraction (GLME) device. Nitrogen served as the carrier gas, with its flow rate precisely controlled at 6 mL/min by a mass flow controller. The extraction temperature was maintained at 20 °C. Following GLME, the extracts were collected in 1.5 mL centrifuge tubes. Without any further pretreatment, these extracts were directly analyzed via DI-ESI-MS.

### 3.3. Optimization of Mass Spectrometry Conditions

A constant flow infusion system was assembled using a precision syringe (100 μL; Hamilton, Bonaduz, Switzerland) coupled to a syringe pump (LSP-01-3A; Longer Pump, Baoding, China). The extraction solution was continuously infused into an Orbitrap Exploris 240 mass spectrometer (Thermo Fisher Scientific, Waltham, MA, USA) equipped with an electrospray ionization (ESI) source for analysis. To achieve optimal ionization and signal response, key mass spectrometry parameters were optimized by monitoring the signal intensity of characteristic marker compounds. The parameters subjected to optimization included the injection pump flow rate (2–7 μL/min), the ion spray voltage (2.5–5 kV), the sheath gas (5–50 arb), the aux gas (6–11 arb), and the ion transfer tube temperature (300–350 °C).

### 3.4. Orbitrap MS Analysis

Mass spectrometric analysis was conducted with an Orbitrap Exploris 240 mass spectrometer (Thermo Fisher Scientific) equipped with an ESI source operated in positive ion mode (+3500 V). The ion transfer tube temperature was set to 330 °C, the *m*/*z* scan range was 600–1000 Da, and the RF lens was set to 70 [51]. Each sample was analyzed in triplicate by direct infusion via an ESI source, using a sample volume of 100 μL and a precise syringe pump flow rate of 4.0 μL/min. Data acquisition was performed using both full MS and data-dependent MS^2^ scan modes. The full MS resolution was set to 60,000, and the collision energy was 40 eV. All raw mass spectral data were processed using Xcalibur 4.2 software. The putative identification of MS/MS fragments was based on comparison with reference data from the literature and online databases (e.g., PubChem, HMDB) [52]. The matching was performed with a mass accuracy threshold of 5 ppm.

## 4. Conclusions

This study developed an integrated strategy combining GLME with mass spectrometry for the targeted enrichment and detection of DDAs in *Aconitum* herbs. The proposed method enables efficient and selective extraction of trace DDAs from complex matrices, permitting direct analysis via DI-ESI-MS without requiring additional sample pretreatment. More importantly, the signal intensities for trace toxic DDAs were enhanced by over 1000-fold in complex *Aconitum* herbs. Furthermore, this approach significantly reduces organic solvent consumption, offering notable advantages in simplicity, efficiency, cost-effectiveness, and environmental friendliness.

The results indicated that raw Caowu exhibited the highest DDA response intensity among the tested samples. Concurrently, considerable DDA signals were still detectable in the three processed products, suggesting that current processing protocols require further optimization to maximize toxicity reduction while preserving pharmacological efficacy. Furthermore, lipo-alkaloids, characterized by their lower toxicity and higher bioactivity, warrant further investigation. Future processing strategies should aim to more efficiently degrade toxic DDAs to achieve the objective of “reducing toxicity while preserving efficacy” or even “reducing toxicity while enhancing efficacy.” In summary, this study establishes an efficient, green, and economical strategy for the analysis of trace toxic DDAs in Aconitum herbs and demonstrates the potential of GLME for the selective enrichment of trace target analytes.

It should be noted that the lack of full quantitative validation of the method renders the methodology unsuitable for routine use in risk assessment. This is partly because the focus of this study was on qualitative structural elucidation and partly because commercial reference standards are difficult to obtain for most of the 27 DDAs. Therefore, if systematic quantitative analysis and accurate toxicity assessment of the observed DDAs are required, systematic phytochemical separation and comprehensive quantitative method validation are recommended first. Additionally, the direct infusion MS (DI-ESI-MS) used in this study inevitably introduces matrix interference. Non-target compounds may cause ion suppression by competing for charges with the analytes, ultimately reducing the MS signal and affecting the quantitative accuracy of the target analytes. Fortunately, this study focused on the qualitative analysis of compounds. The results indicate that the changes in relative signal intensity potentially caused by matrix interference do not significantly affect the acquisition of MS/MS data for target analytes and do not hinder structural identification. Thus, considering multiple factors such as analysis time, DI-ESI-MS was employed to characterize samples collected via GLME. Finally, current international research on GLME primarily remains at the level of phenomenological description and application, with its specific mechanisms of action still subject to debate. The 27 DDAs identified in this study as being efficiently enriched by GLME provide highly representative examples for delving into the intrinsic relationship between GLME enrichment efficiency and compound structure and physicochemical properties. This will be a very important and interesting study to carry out in future research.

## Data Availability

The original contributions presented in this study are included in the article/Supplementary Materials. Further inquiries can be directed to the corresponding authors.

## Supplementary Materials

The following supporting information can be downloaded at https://www.mdpi.com/article/10.3390/molecules30194029/s1: Figure S1: The structures of 27 compounds were identified by compound standard, reference, and online compound databases; Figure S2: The MS/MS spectra of 11 known alkaloids identified by references and online databases; Figure S3: The MS/MS spectra of 10 known lipid alkaloids identified by references and online databases; Table S1: Comparison of response intensity of three main diester diterpenoid alkaloids before and after GLME; Table S2: Structures and main information properties of the 3 key compounds.
