# Supplementary material for: Targeted Enrichment and Characterization of Diester Diterpenoid Alkaloids in Aconitum Herbs Using Gas–Liquid Microextraction Coupled with High-Resolution Mass Spectrometry
Source: Molecules. 2025 Oct 9;30(19):4029. doi: 10.3390/molecules30194029 (PMC12525639; doi:10.3390/molecules30194029)
Supplement: Supplementary file 1 [file molecules-30-04029-s001.zip › molecules-3878225-supplementary.pdf]

Supplementary Materials for:

Targeted Enrichment and Characterization of Diester Diterpenoid Alkaloids in *Aconitum* Herbs Using Gas–Liquid Microextraction Coupled with High-Resolution Mass Spectrometry

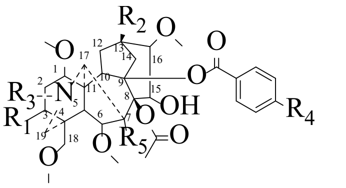

1 R_1=R_2=R_4=R_5=H, R_3=CH_3
2 R_1=R_3=R_4=R_5=H, R_2=OH
3 R_1=R_2=R_4=R_5=H, R_3=CH_2CH_3
4 R_1=R_4=R_5=H, R_2=OH, R_3=CH_3
5 R_1=R_4=R_5=H, R_2=OH, R_3=CH_2CH_3
6 R_1=R_2=OH, R_3=CH_3, R_4=R_5=H
7 R_1=R_2=R_5=H, R_3=CH_2CH_3, R_4=OCH_3
8 R_1=R_2=OH, R_3=CH_2CH_3, R_4=R_5=H
9 R_1=R_2=R_5=OH, R_3=CH_3, R_4=H
10 R_1=OH, R_2=R_5=H, R_3=CH_2CH_3, R_4=OCH_3
11 R_1=R_2=R_5=OH, R_3=CH_2CH_3, R_4=H

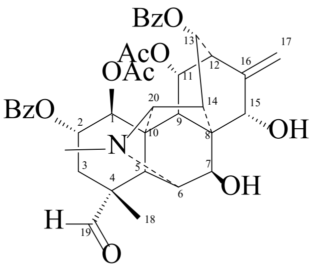

15

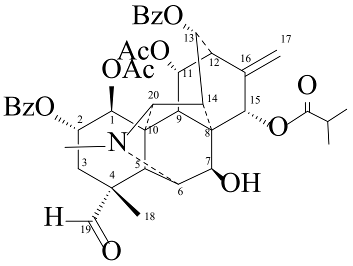

16

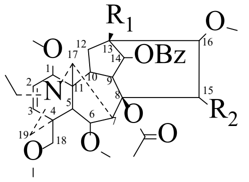

12 R_1=R_2=OH
13 R_1=H, R_2=CH_2CH_3
14 R_1=OH, R_2=CH_2CH_3

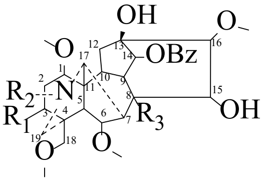

17 R_1=H, R_2=CH_2CH_3, R_3= pal
18 R_1=OH, R_2=CH_3, R_3= pal
19 R_1=H, R_2=CH_3, R_3=lino
20 R_1=H, R_2=CH_3, R_3=lino
21 R_1=OH, R_2=CH_2CH_3, R_3=pal
22 R_1=H, R_2=CH_2CH_3, R_3=lino
23 R_1=H, R_2=CH_2CH_3, R_3=lino
24 R_1=OH, R_2=CH_3, R_3=lino
25 R_1=OH, R_2=CH_2CH_3, R_3=lino
26 R_1=H, R_2=CH_3, R_3=pal
27 R_1=OH, R_2=CH_3, R_3= eico

Figure S1 The structures of 27 compounds were identified by compound standard, reference and online compound database. (pal, lino and eico in figures represent the residues of palmitic acid, linoleic acid, eicosapentaenoic acid, respectively)

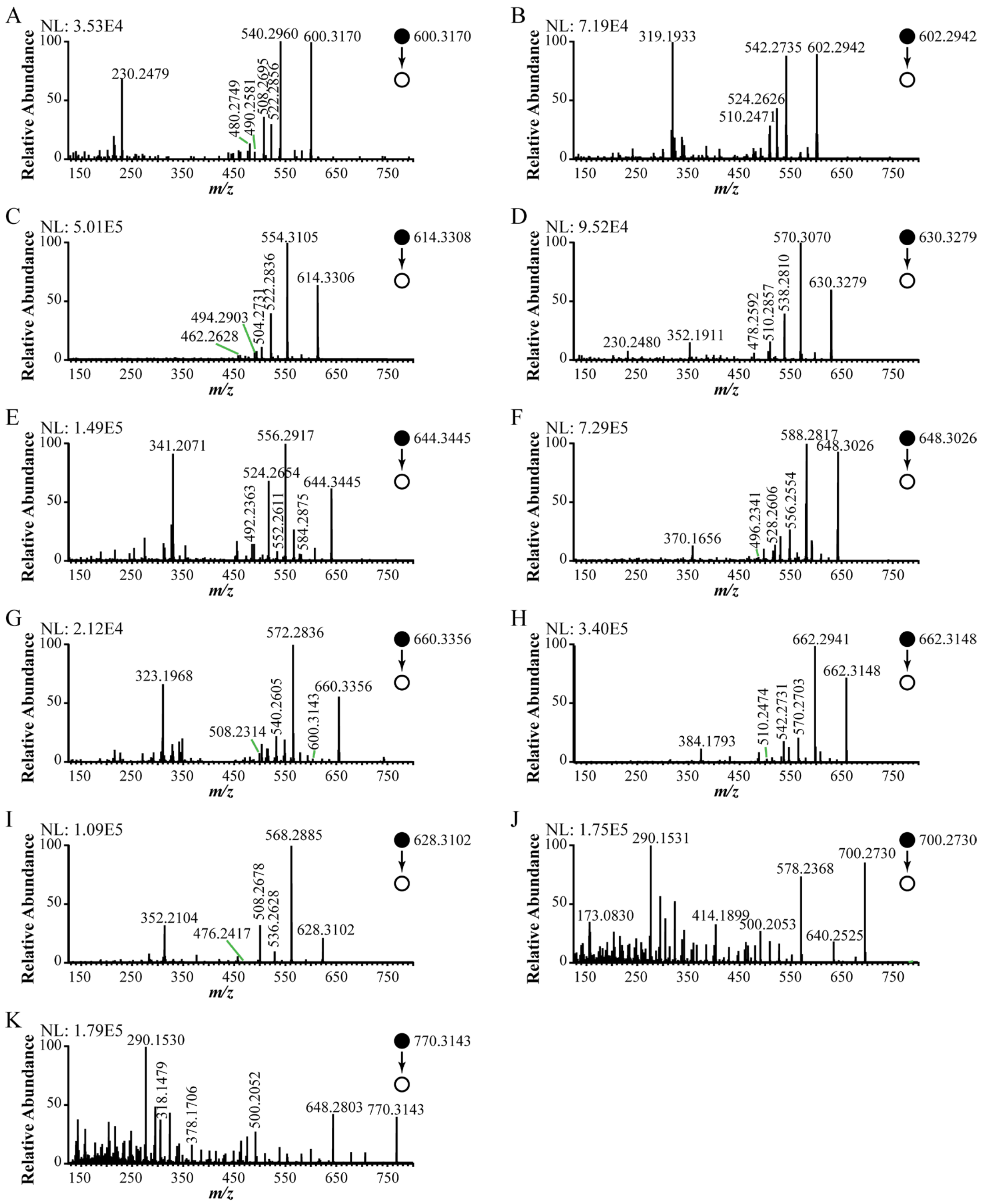

Figure S2 The MS / MS spectra of 11 known alkaloids identified by references and online databases. (A Delphinine, B N-demethylhypaconitine, C Chasmaconitine, D 3-Deoxyaconitine, E Crassicauline A, F Beiwutine, G Yunaconitine, H 10-Hydroxy aconitine, I Anhydroaconitine, J Trifoliolasine E, K (-)-1β,11a-diacetoxy-2α,13α-dibenzoyloxy-7β-hydroxy-15α-isobutanoyloxy-N-methyl-N,19-secohetisan-19-al)

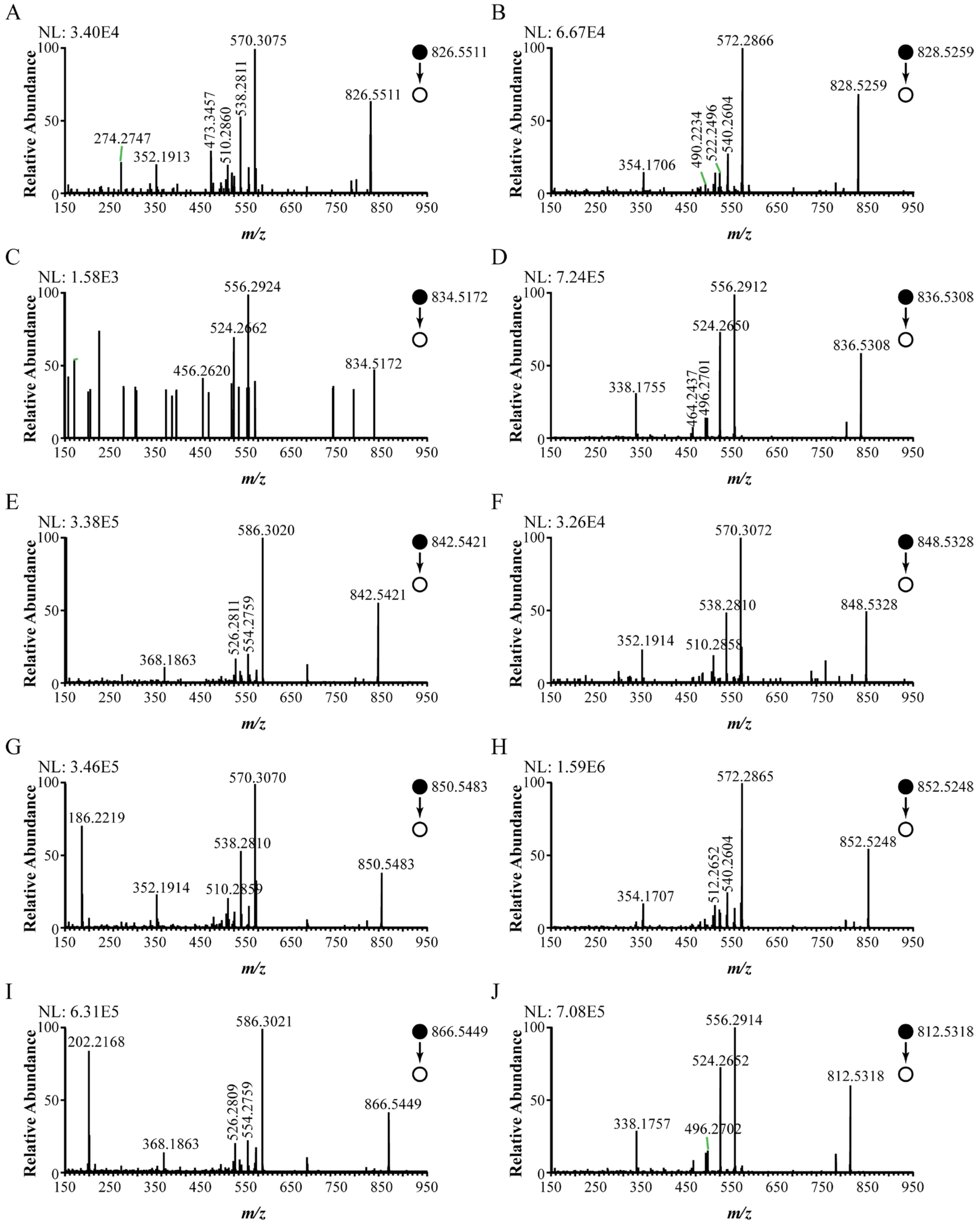

Figure S3 The MS / MS spectra of 10 known lipid alkaloids identified by references and online databases.(A 8-palmitic-benzoyldeoxyaconitine, B 8-palmitic-benzoylmesaconine, C 8-linolenic-benzoylhypaconine, D 8-linoleic-benzoylhypaconine, E 8-palmitic-benzoylaconine, F 8-linolenic-benzoyldeoxyaconitine, G 8-linoleic-benzoyldeoxyaconitine, H 8-palmitic-benzoylmesaconine, I 8-linoleic-benzoylaconine, J 8-palmitic-benzoylaconine)

Table S1 Comparison of response intensity of three main diester-diterpenoid alkaloids before and after gas-liquid microextraction

**Samples**	**Hypaconitine**		**Mesaconitine**		**Aconitine**	
	Before	After	Before	After	Before	After
**Raw Fuzi**	3.26E+04	1.37E+07	4.91E+03	4.57E+06	2.80E+04	1.47E+06
**Raw Caowu**	2.98E+03	2.35E+07	4.19E+04	1.97E+07	9.52E+03	8.54E+06
**Raw Chuanwu**	4.74E+03	1.72E+07	1.81E+03	1.40E+07	-	5.68E+06
**Processed Fuzi**	6.84E+03	7.94E+06	2.31E+03	5.17E+05	-	9.72E+05
**Processed Caowu**	1.16E+03	6.73E+06	6.27E+03	3.76E+06	-	2.40E+06
**Processed Chuanwu**	6.93E+03	9.65E+06	2.29E+03	2.73E+06	-	2.61E+06

Table S2 Structures and main information properties of the 3 key compounds.

**Analytes**	**Aconitine**	**Mesaconitine**	**Hypaconitine**
Structure	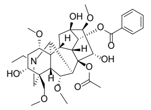	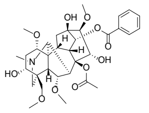	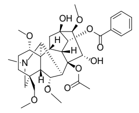
CAS	302-27-2	2752-64-9	6900-87-4
Formula	$C_{34}H_{47}NO_{11}$	$C_{33}H_{45}NO_{11}$	$C_{33}H_{45}NO_{10}$
HPLC	≥ 98%	≥ 98%	≥ 98%
Lot Number	20071702	23120319	2108601
